# Restitution of Sculptural Groups Using 3D Scanners

**DOI:** 10.3390/s110908497

**Published:** 2011-09-01

**Authors:** Pilar Merchán, Santiago Salamanca, Antonio Adán

**Affiliations:** 1 Escuela de Ingenierías Industriales, Avda. de Elvas, s/n, Universidad de Extremadura, 06071 Badajoz, Spain; E-Mail: ssalamanca@unex.es; 2 Escuela Superior de Informática, Ronda de Calatrava, 5. Universidad de Castilla La Mancha, 13071 Ciudad Real, Spain; E-Mail: Antonio.Adan@uclm.es

**Keywords:** 3D scanning, cultural heritage, virtual modelling

## Abstract

Imagine for a moment that you have to solve a 3D jigsaw of which you have lost several pieces. You have also lost the original box-top showing the final picture, and as if that were not enough, some of the pieces you do have may belong to some other jigsaw. This is in essence the sort of challenge that we faced in the novel project that we shall be describing in this paper. The final aim of the project was, with the help of 3D scanners, to digitalize and reconstruct multi-piece classical sculptures. Particularly, we tackle the restitution of the so-called “Aeneas Group”, a famous iconographic reference during the Roman Empire. We have undertaken this ambitious project in collaboration with the research department of the Spanish National Museum of Roman Art (MNAR). This paper summarizes the real problems that arose and had to be solved, the innovations, and the main results of the work that we have carried out over these recent years.

## 1. Introduction

A wide variety of non 3D-visual techniques and sensors have been introduced in the last years in many processes associated with obtaining, preserving and archiving archaeological remains. Examples of those techniques can be found in [1] (near-infrared (NIR) sensors), [2] (contrast and visual enhancement techniques), [3] (chemical sensors), [4] (laser induced breakdown spectroscopy (LIBS)), [5] (gas-chromatographic analysis) and [6] (hyperspectral reflectance). However, visual sensors and 3D scanners (laser and light structured sensors) have usually played the main role in this framework over the last years.

Usually 3D laser scanners are classified in three types: Triangulation based scanners, Time of flight scanners and Phase-Shift scanners. All of them can be used in archaeology for different environments and requirements. In this paper, we present a restitution work using a triangulation light block method. This device is composed of a camera and a laser projector which sweeps the scene. The system processes multiple images of the scene with the projected laser line and yields the 3D information. In general, the set of processes related to obtaining the raw data provided by the 3D scanners is called *3D scanning* and the work needed to transform these raw data into models is called *3D modeling*. In our case these models are use for archaeological purposes. An interesting discussion on the state of the art in 3D imaging in cultural heritage and other applications can be consulted [7].

The reason why 3D scanners are widely used in the cultural heritage field is that the requirements of cultural heritage applications-high precision, dense sampling and preserving the integrity of the surfaces-make them the best technological choice. The availability of an accurate digital representation opens up a broad spectrum of possibilities for experts. 3D models are also the starting point for the design of many applications aimed at disseminating cultural heritage which are based on visual encoding and communication. They range from the interactive/immersive applications (virtual and augmented reality, haptic immersions…) to the more passive ones (still images, videos, computer animations, digital reproductions…).

There are numerous benefits that make virtual restoration projects attractive: the generation of documental databases of heritage pieces so that they can be reconstructed or restored in the case of fire, earthquake, flood or wind erosion, providing historians and archaeologists exact copies of the original pieces; the interaction with precise physical replicas avoiding moving and touching the original pieces which would entail the risk of damage or contamination; the creation of educational resources for researchers and students of history and art, which make works of art accessible to the public and facilitate virtual museum exhibitions and virtual tourism. The kind of information that has to be handled also makes this framework an exceptional testing ground for new algorithms of 3D data visualization and processing. Last, but certainly not least, the mutually enriching formation of multidisciplinary teams is also a challenging aspect to bear in mind.

Digital data of heritage objects can be obtained using modern computer vision techniques. In cultural heritage restoration, 3D scanning results have until now served primarily to produce still images, interactive visualizations, or animations. The principal applications have been oriented to faithful rendering of objects for their physical reproduction by rapid-prototyping technology. Beyond the initial fascination produced by the beauty of the resulting reproductions, questions inevitably arose about how best to use the 3D models. While there have been various studies using 3D graphics as an analytical tool [8–13], in general projects of this type are still rare.

The work that we shall describe in this article belongs to this rather select group, at least in terms of numbers. It consisted of acquiring digital data with which to build a 3D model of the famous Roman Empire sculptural group known as the Aeneas Group. Our goal was not just to make a set of statuary fragments legible with newly available technologies and to produce a model of the group, but also to provide historians with tools and materials that would enable them to ask and seek answers to their own research questions. Our ultimate aim was the reconstitution of the masterpiece in the sense that we would be able to allocate all of the fragments to their correct position in a virtual model.

**Figure 1.**
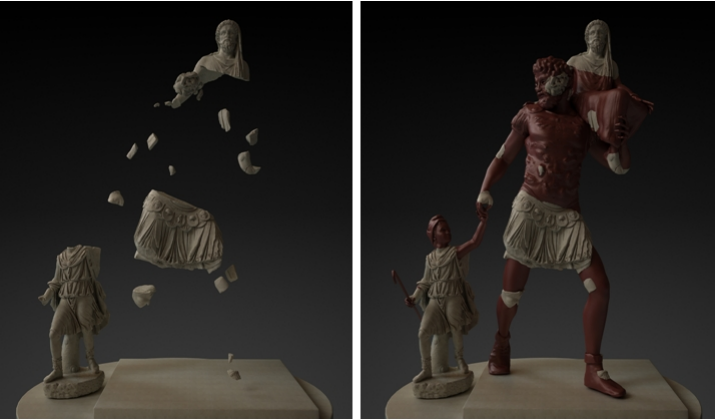
Virtual restitution proposed in Section 4.1. **Left:** Placement of the 30 recovered fragments. **Right:** the fragments in their proposed position integrated on a virtual model of the masterpiece.

Reconstruction of archaeological pieces from fragments is an arduous and difficult task if it is manually performed. For this reason, the automatic reconstruction of fragmented objects through the matching of its fragments is being currently researched in archaeology, palaeontology and art restoration. It is crucial to find methods that allow executing these tasks by help of a computer. This objective can be accomplished by the analysis of some object characteristics such as: colour, texture, shape, and some others. The main problem we faced in this work for the restitution of this sculptural group was that we only have 30 pieces, most of them of small size, that obviously lead to a final solution with lots of gaps (see Figure 1). The exact collocation of all the pieces is clearly a far from straightforward problem, but finding a solution would have worldwide repercussions since the restitution of the Augusta Emerita group would also imply the restitution of the group of the Imperial Forum of Augustus in Rome.

This paper starts with a brief presentation of the Aeneas group with the purpose of making its importance within the Roman Empire quite clear. It continues with the description of methodological questions related to the scanning system itself and the procedure chosen for the correct digitalization of the findings. First, the stages followed for the data acquisition phase and the particular needs shown by this kind of objects are presented. Then, it deals with the geometrical reconstruction of the scanned pieces. Next we highlight the most important aspects of the color integration task. These considerations are followed by a description of our solution for the positions of the set of recovered fragments on a virtual representation of the complete Aeneas group, which was the main purpose of this work. A final discussion on conclusions and future works closes the paper.

### 1.1. The Aeneas Group

*Augusta Emerita*, the capital of the ancient Roman province of *Lusitania* in *Hispania*, became one of the most representative centres of Romanization of the Iberian Peninsula soon after its founding in the year 25 BC. In 1993, this archaeological site, located in the city of Mérida, capital of the province of Badajoz in west-central Spain, was designated a World Heritage Centre by the UNESCO.

**Figure 2.**
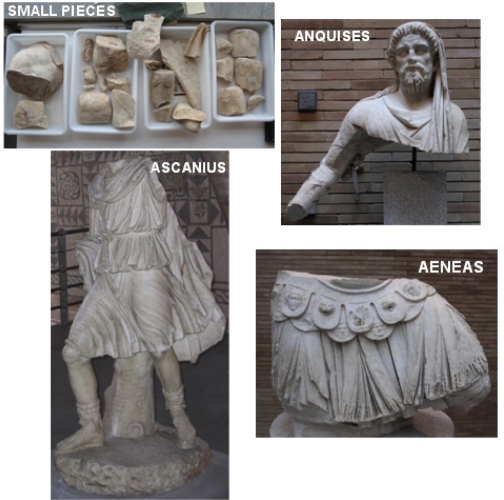
Pieces found in the excavations of the Forum of Augusta Emerita.

Among the pieces recovered during the 19th Century excavations of the *Forum* of *Augusta Emerita* was a statue which, because of its typology, was at first believed to be of the huntress Diana. Later, however, it was identified as Ascanius, son of Aeneas. In the 1980s, two large statuary pieces were found: the lower part of the breastplate and the beginning of the legs of a larger-than-life-size male figure, and the upper third of another male figure–the bearded head and veiled body of a middle-aged person. About 25 small fragments from the same period have now been found (Figure 2).

**Figure 3.**
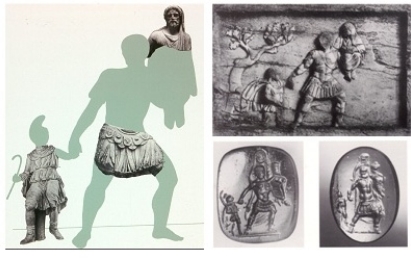
**Left:** Reconstitution of the Aeneas Group according to Trillmich and Nogales. Reproduction of the Aeneas Group on a relief (**upper right**), and two seals (**lower right**).

The fundamental studies of Prof. Trillmich on the successive sculptural findings established the connection between these pieces and the hypothetical reconstitution of a single group belonging to the Spanish complex which would have been a provincial replica of the Aeneas Group in the *Forum Augustum* of Rome, known only from documentary and iconographic references [14]. The Aeneas Group consisted of three complete human pieces that had an overall size of around 6.5 cubic metres. It represented the figure of Aeneas escaping from Troy, holding his son Ascanius by the hand, and carrying his father Anchises on his shoulder (Figure 3).

## 2. Scanning System and Methodology

**Figure 4.**
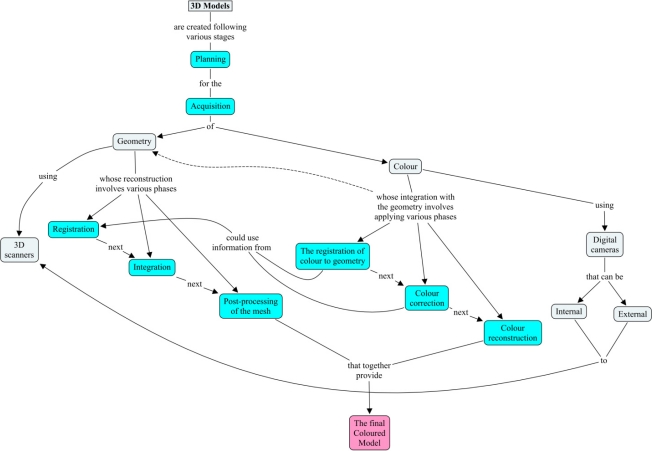
Conceptual map of the reconstruction of a model from multiple overlapping scans.

Digitalization of Roman sculptures poses many problems. Roman characters often wear pleated tunics or robes, so that there are many regions which are inaccessible to the scanner. The sculptures are of marble, with the resulting problem of reflections and gleams when the scan is taken, leading to frequent errors in the 3D data and corrupted colours. In order to properly scan the entire piece, one must use elevator platforms. Finally, the massive amount of information in the hundreds of views has to be matched. Our 3D reconstruction method follows the typical stages of the 3D modelling process [15]. Figure 4 shows a conceptual map of the whole procedure.

As known, within a conceptual map, concepts are represented as boxes connected with labeled arrows in a downward-branching hierarchical structure and the relationship between them is articulated in linking phrases. Modelling essentially consists of three processing stages, each divided into various steps. These processes are the concepts which are shaded in blue in the map of Figure 4 for ease of identification. The initial stage consists of planning and data acquisition. Following this, the processing is split into two streams: one for the geometry, and one for the fine-scale surface appearance—the texture. Information can be exchanged between these two streams to improve the quality and efficiency of how each data type is processed. In the map, this exchange of information is depicted by links between the concepts of the two main streams. In the final stage, the geometry and colour properties are combined into a single compact numerical description of the object—the final coloured mesh.

### 2.1. Data Acquisition

Data acquisition planning is a fundamental stage in 3D digitalization processes, but this planning is particularly important in the case of the reconstruction of cultural heritage since a loss of data due to poor planning might be hardly recovered.

In addition, scanning a large statue in a museum imposes a number of constraints on the design of the scanning system and the procedures to follow. In our case, the irreplaceable nature of the pieces meant that contact had to be restricted to a minimum, and required us to ensure that we operated the scanning system safely. Because of the complex shapes of the figures, we had to be able to freely position the sensor so as to access the recessed parts of their surfaces.

That is why we decided to use a system that could capture a multitude of small portions of the surface from different set positions of the scanner. The resulting large number of overlapping scans would then be seamlessly integrated using specific registration software. The scanner used was a Minolta Vivid 910. This device provides good quality and accuracy for the type and size of the fragments to be digitalized. Thus, 640 × 480 individual points can be measured per scan, furthermore it requires only 0.3 s for data input and when operating in fine mode, precision of ±0.008 mm and accuracy of ±0.10 mm can be achieved on the Z-axis. Under our scanning conditions, the area covered by each scan depended on the lens used and the sensor-object distance. Table 1 gives the camera parameters used to digitize the large pieces of the Aeneas Group, illustrating the relationship between the lens used in the sensor, the sensor-object distance, and the area covered by each scan. The longer the lens’ focal length, the smaller the total surface area captured, but then of course the greater the detail in digitizing the zone. In the case of the Aeneas character, for instance, two lenses were employed. The *middle* lens was used to acquire most of the surface except for the belt area, where the relief made it necessary to use the *tele* lens (see the photograph of Aeneas in Figure 3). The specifications concerning the level of detail were set above 20 points per square millimeter and 4 patches per square millimeter.

**Figure 5.**
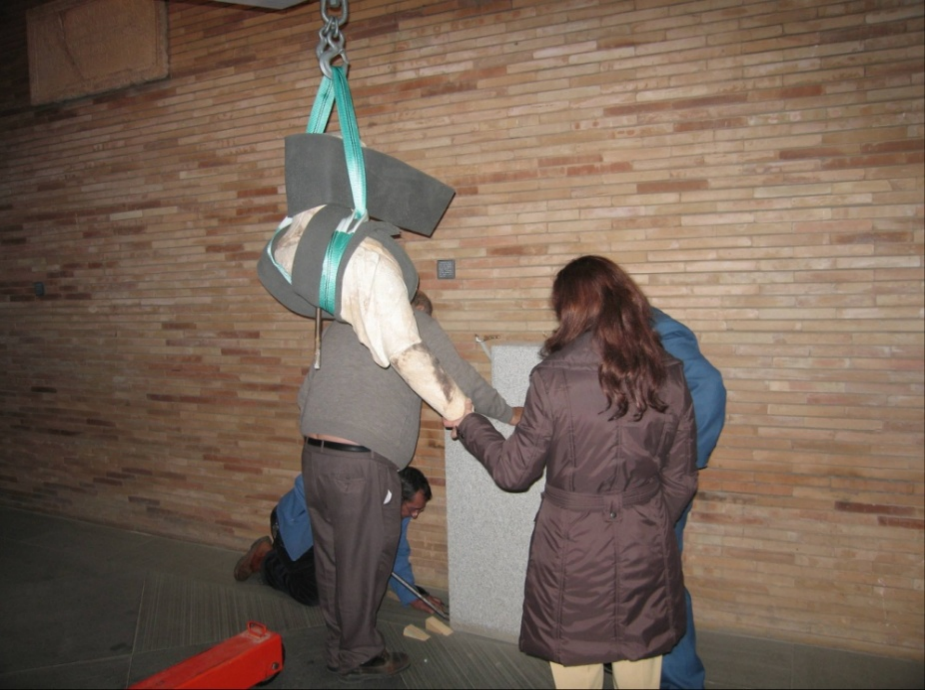
The Anchises fragment being lifted with a crane to turn it the other way around so we could scan the back area.

**Figure 6.**
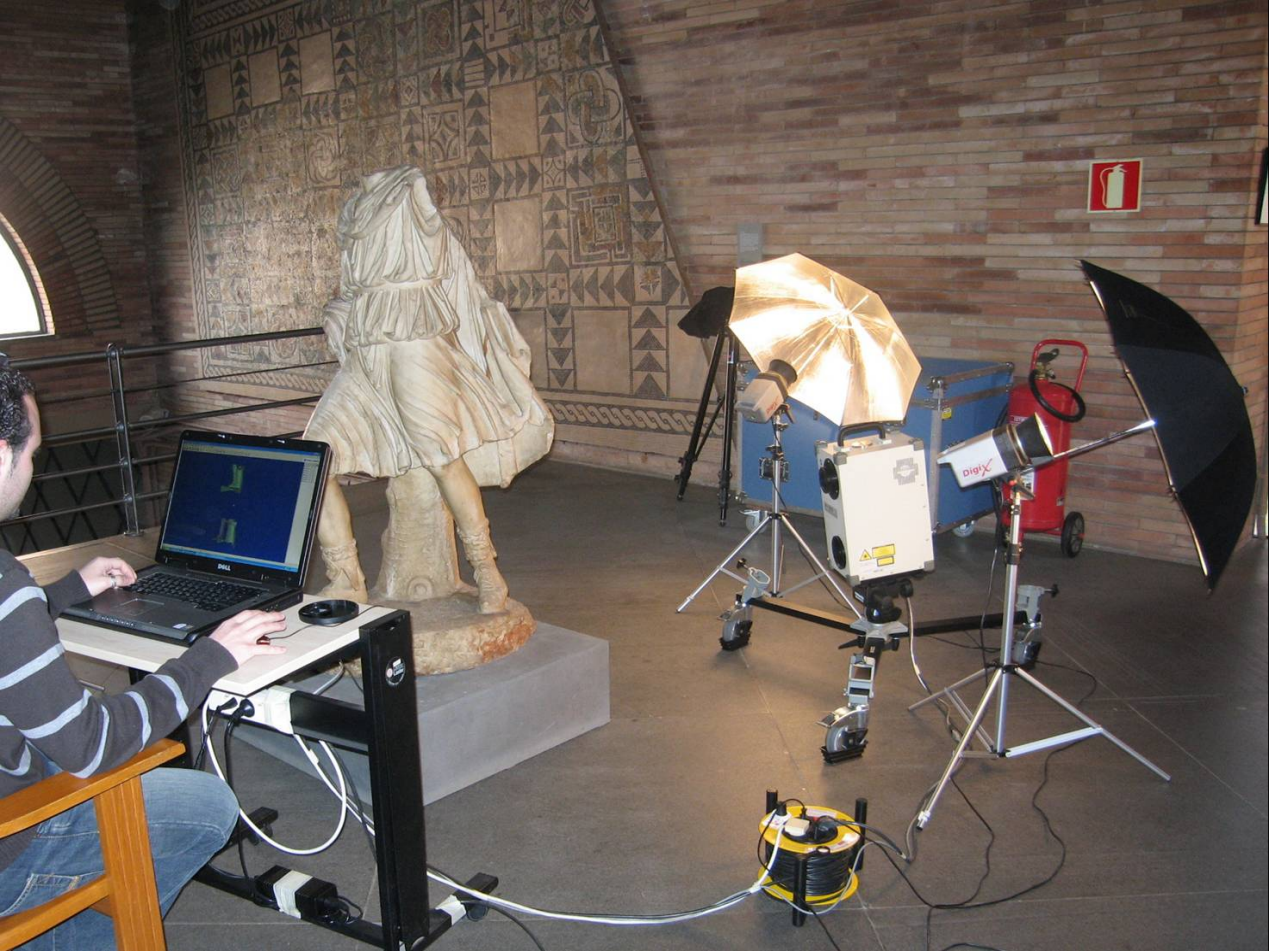
Moment of the scanning of Ascanius in the MNAR. The picture shows the lighting system used and the scanner mounted on a dolly.

To scan the 25 smaller fragments, we used a rotation table. We thus obtained automatically registered views of the object which were uniformly separated over a complete 360° turn. The figure of Anchises had to be hoisted with a crane and turned to allow us to acquire the back area data. The photographs in Figures 5 and 6 show various moments of the data acquisition process. This stage was carried out in four expeditions of approximately one week, spending five or six full days in the museum on each visit.

### 2.2. Geometric Modelling

The geometrical reconstruction of the scanned pieces from the numerous partial views we took was done in three steps. The entire data processing section was carried out by using internally developed (non-commercial) software.

The first stage was the registration of the partial views of the object in a common reference system. As is usual, registration is implemented through geometrical transformations (rotation and translation). The computational techniques used in this process were the kd-tree algorithm and point-to-point minimization [16], a variation of the well-known ICP registration technique.

The second step was to integrate the multiple aligned data into a single complete mesh model representing the entire surface of the object. This step is referred to as ‘*merging*’. In essence, the procedure determines a set of single surface positions from multiple overlapping surface observations. In this merging procedure, it is important to make the integration framework robust against any noise which is present in the scanned images or which may be inherited from the registration process. The merging stage was carried out by following the well known mesh zippering algorithm [17]. After this process, the output is a model mesh *M* fitted to the whole surface of the object.

In the third stage, the single mesh obtained, *M*, is subjected to a hole-filling algorithm. The technique that we implemented is based on an image inpainting algorithm that uses the *Fields of Experts* [18] framework. Previous approaches to this problem had lacked general validity in the sense that they were designed to provide good solutions for some specific kind of holes in 3D meshes, but not for a wide range of cases. For this reason, we decided to test a Fields of Experts algorithm due to the excellent results these techniques provide for a large variety of situations. This method can be summarized in the following steps:

1. Holes identification in *M.* These holes are denoted as *h_i_* *(i = 1, ..., m)*;
2. Selection of a particular hole *h_i_*;
3. Definition of the portion of the mesh *M_υ_i__ ⊂ M*, which is the part of the mesh in the surroundings of *h_i_*;
4. 3D to 2D transformation to obtain the range image, *I_h_i__*, related to *M_υ_i__*;
5. *Image inpainting* algorithm application on *I_h_i__*. The resultant image is *I_f_i__*;
6. 2D to 3D transformation of the new data generated by the *image inpainting* algorithm. In order to have a mesh without the hole *h_i_*, the new mesh, *M_f_i__*, is merged with *M*;
7. Repeat from step 2 with the next hole identified in *M*, *h*_i+1_.

**Figure 7.**
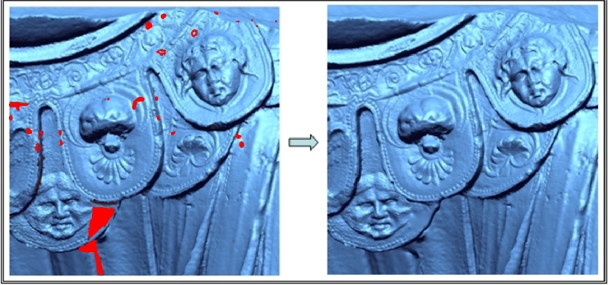
Details of the results of the hole-filling technique put into practice on the Aeneas belt.

Extensive experimentation with our algorithm demonstrated its robustness in suitably filling holes of any size. Initial work can be found in [19]. This paper describes a method for filling holes in a 3D partial mesh, which corresponds to only one view of the object. The method presented here is an extension of that work in which the filling hole for a complete mesh model is resolved. Figure 7 illustrates the result of applying this technique to several holes in Aeneas's belt in the geometrical model obtained after the final merge. Holes are depicted in red.

### 2.3. Colour Integration

The geometrical model provides essential information of an object, because it allows us to analyze that object in detail. But, in addition to the geometric data, the surface colour distribution (texture) is also very important for some kinds of cultural properties. The process of colour reconstruction plays therefore a key role in the course of integral reconstruction.

The textural information we used was provided by the scanner itself. We shall call this colour map a ‘colour image’. For each scan, every point of the sensed surface has an associated pixel in the colour image. We can thus establish the correspondence between geometry and color in each view and the process of assigning the color to the 3D points for each scan can be easily solved. However, the integrated model will have multiple local errors, and the different scans of overlapping samples will have different colours assigned to the pixels. This is because the integration of the different colors acquired from different views is still an open problem in computer vision research. Several partial solutions have been proposed to achieve high-quality colour integration. Most are based either on global approaches in which the complete texture is processed in a single step (and then modified), or on local strategies in which only the colours of some points of the image are varied. Recently, Callieri *et al.* have proposed a multivariate blending function which weights the colours assigned from different viewpoints taking geometric, topological and colorimetric criteria [20]. The weighted colours are then selectively mapped on the mesh model. In another interesting work (Corsini *et al.* [21]) the authors use mutual information between the image to be registered and renderings of the model geometry in order to make the colour integration. They follow an iterative process.

We had two strategies to achieve the total colour integration: processing all the partial images in parallel in a single step, or implementing a procedure of sequential integration. We chose the sequential procedure mainly for two reasons: it allowed us to monitor the colour merging process and to check the result as each new view is added, and the algorithmic and computational requirements were far less complicated than in a parallel strategy.

Our approach combines some of the best aspects of global and local strategies. The colour integration proposed is a sequential off-line process which starts by mapping the colour of the first scan onto the complete mesh model of the object. The colour model grows as new view is added. From here on we will name this as “current colour model”. When a new view is added to the current colour model, we apply an integration algorithm between the current colour model and the new colour information, which might corresponds (or might not) to nodes which have already an assigned colour in the current colour model. As a result of this process, the current colour model is updated. More information can be found in [22].

**Figure 8.**
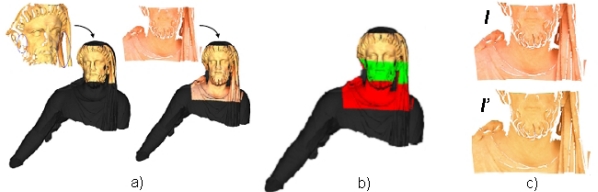
(**a**) Sequential procedure: Partial colour mapping superimposed onto the geometrical model. (**b**) The entire geometrical model with the intersection region in green. (**c**) The view to be merged before and after the initial correction.

The colour integration algorithm consisted of three stages: initial correction, colour merging and refinement. In the initial correction phase, the colour image of the next view to merge in the sequence (which we will call *I*) is corrected taking into account its region of intersection with the current integrated texture (which we will call *T*). Since the colour changes in the objects we were dealing with (marble pieces) were smooth, we used a linear transformation for the relationship between the colours corresponding to the points in the intersection region. Applying this transformation over *I*, we obtained a corrected colour image *I’*. We can say that, in this stage, a global strategy which modifies all pixels of *I* is applied. Figure 8 illustrates this sequential procedure: Figure 8(a) is an example of the sequential integration and illustrates the partial colour mapping superimposed on the geometric model; Figure 8(b) shows the entire geometrical model and the region of intersection between the colour images assigned to the model after *n* views have been integrated (*T*) and the image corresponding to the next view to be merged (*I*) in green; and Figure 8(c) compares the view to be merged before and after the global correction. In the colour merging phase, the RGB components of the overlapping points in the two colour images, *T* and *I’*, are subjected to a weighted-mean transformation. The weights assigned to the colours depend to the viewing ray and the surface normal in the complete model and the new scan. We can thus say that, in this stage, a local strategy is taken. Finally, the refinement phase applies two types of filter to the colour images. The discontinuities that typically arise at the seam or edge points after the local correction are resolved using a 3D linear smoothing filter, and a 3D discrete Gaussian filter is applied to the three colour components.

**Figure 9.**
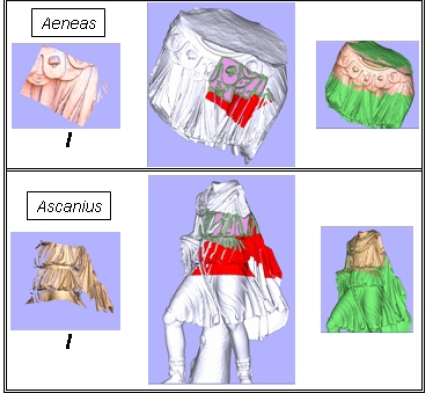
**Left:** Images to be merged (*I*) from the Aeneas and Ascanius’ scans. **Centre:** Filter windows superimposed onto the models, the seam patches are shaded in grey and the points that fall into the Gaussian mask in green. The pink zones represent redundant colour. **Right:** the final result after the final smoothing phase.

The left side of Figure 9 shows two images to be merged (*I*). In the two central pictures, the seam patches are shaded in grey on the geometrical models, and the points that fall into the Gaussian mask in green. The pink zones represent redundant colour in the sense that it is information from *I* which is already in the current integrated textured, *T.* The red zones represent new colour provided by *I*. The two pictures on the right of the figure show the result after this final smoothing phase.

## 3. Digital Models

**Figure 10.**
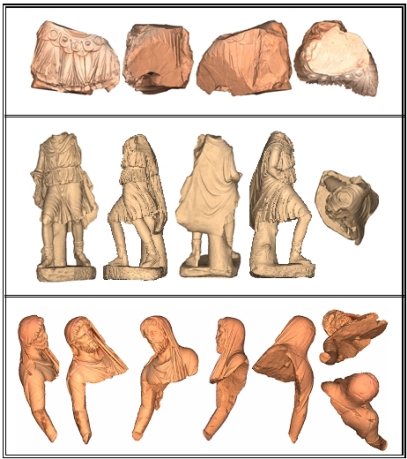
Final models for the three biggest pieces.

**Figure 11.**
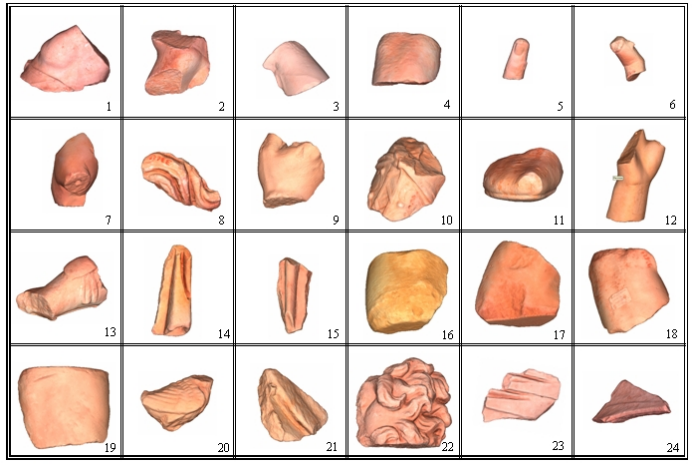
Final models for the small fragments.

A complete digital model has been produced for each of the recovered fragments. They are shown in Figures 10 and 11. The information regarding the final geometrical models are summarized in Tables 2 and 3. Table 2 concerns data of the three large pieces models and Table 3 the corresponding to the small segments. In them, the column ‘Views’ gives the total number of scans needed to digitize the piece completely. The next column (‘Faces max.’) gives the number of triangular faces in the most accurate final mesh. The last two columns give the values of the total surface area of the model, and the volume of the bounded box that contains this model.

## 4. Transferring the Results to Historians

### 4.1. Virtual Reconstruction

As we noted in the Introduction, one of the main purposes of this project, and perhaps the most valuable for art historians and archaeologists, was to solve the 3D puzzle of reconstructing the entire Aeneas sculptural group given that the pieces which remain are highly incomplete.

The automated reconstruction of fragmented objects by matching the fragments is currently an active area of research in archaeology, palaeontology, art restoration, *etc*. Although manual reconstruction is possible, the tasks involved are usually both difficult and tedious. It is therefore crucial to find methods that allow these tasks to be carried out with the aid of a computer.

In a six DOF context like this, the coupling of separate fragments into a hypothetical original masterpiece has, to date, remained an unsolved problem. This is owing to the fact that there are two different problems which cannot be solved simultaneously by intelligent techniques. First, a masterpiece should be proposed by the computer, but this is not possible if only a few unconnected 3D fragments are available. Secondly, the membership and pose of these fragments into a proposed masterpiece cannot be inferred by the computer if no reliable original 3D model exists.

**Figure 12.**
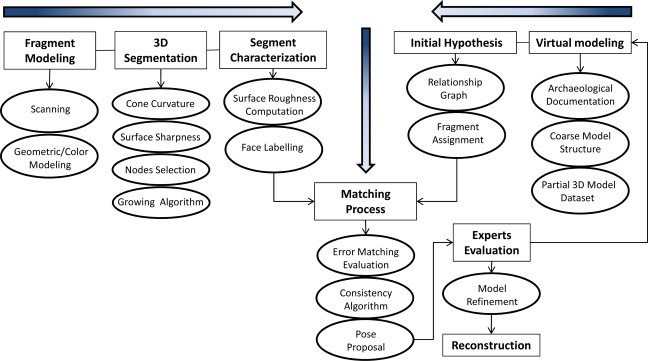
Overview of the virtual reconstruction process.

We used a hybrid human-computer strategy to solve this extremely complex incomplete 3D puzzle problem. The main idea is that an interaction between an archaeological knowledge database-built by historians and archaeologists-, and a computer knowledge database-obtained by computer science experts-, is needed to achieve a reliable solution. In Figure 12 an overview of the hybrid human-computer strategy is presented. We will give an explanation of this chart in the following paragraphs.

**Figure 13.**
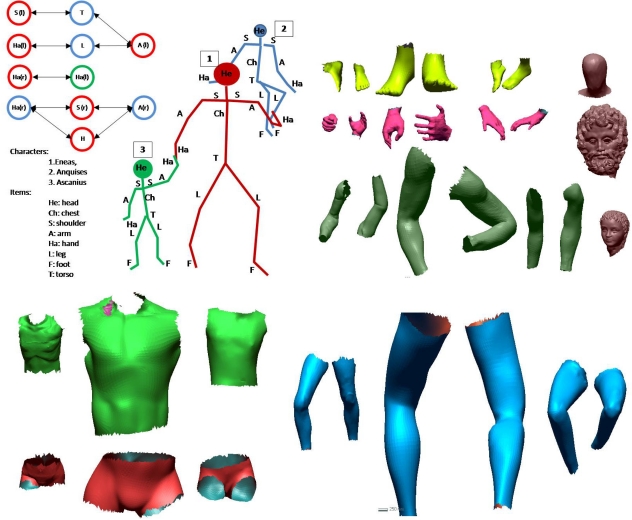
(**a**) Elemental structure of the model proposed including essential parts of the body and the relationship graph. (**b**) Models of some parts of the characters scaled to real size.

The archaeological knowledge database was obtained from the remains found on the site, from many sources such as paintings, coins, small bas-reliefs and texts. Thus a coarse model of the group was consequently proposed. Essentially, the structure of the sculptural group was: two standing figures with joined hands-which correspond to an adult and a child-, and an old man who is carried on the adult’s shoulders. An elemental structure of the model proposed is shown in Figure 13, in which the body is divided in several parts: head, chest, shoulder, arm, hand, leg, foot and torso. Each part of the body of the three figures was then modeled by taking 3D standard human models. Besides, a neighborhood relationship between some parts of different characters was also established by historians. For example, Aeneas’s right hand and Ascanius’s left hand should be in contact. All this information was used by the modeler to build a rough 3D model of the Aeneas group. Figure 13(b) illustrates the models of different parts of the bodies and the neighborhood graph.

In order to obtain a reliable and effective reconstruction, we considered these hypothesis provided by historians and archaeologists along with the assignation of each fragment to a specific part (or a set of parts) of the body. This is not quantitative but qualitative information which reduces the area of the complete virtual model to which the fragment will be matched. For example, a fragment might be labeled as a part of a foot and, the matching process would therefore be solely restricted to “foot” regions in the virtual model.

On the other hand, from the artificial intelligence point of view, the computer was able to infer a set of properties from the fragments and propose an accuracy pose in a 3D virtual model, refining the information provided by historians. In our work, the automatic surface characterization process labels the faces of the pieces as either original or fractured. It is based on a two-step algorithm. In the first step, the pieces of the sculptures are segmented into faces.

The 3D segmentation phase uses the concept of *cone curvature* [23]. With this feature calculated for every node in the triangular mesh of the 3D model, a value of *surface sharpness* is defined. This concept allows us to set which parts of the model mesh are edges and which are not. To do this, we first select the nodes in which *surface sharpness* overcomes a threshold and then obtain the triangular patches with all three vertices selected. This resulting set of triangles determines a coarse region in the 3D model where the edges of the fragment will later be found. After the edge-region is extracted, a growing algorithm is carried out by taking any non-edge node as a seed.

The segmented faces identified in the previous step are then labelled as either *original* or *fractured*. To do this, we define the feature *roughness* attached to each node of the face to be characterized. This feature is calculated with the variance of the normals in the neighbourhood of the chosen node. By means of the comparison between the roughness probability density function for a given zone and the learned probability density functions, the segmented face is eventually labelled either as *rough* (that is, *fractured*) or *non rough (original)*.

**Figure 14.**
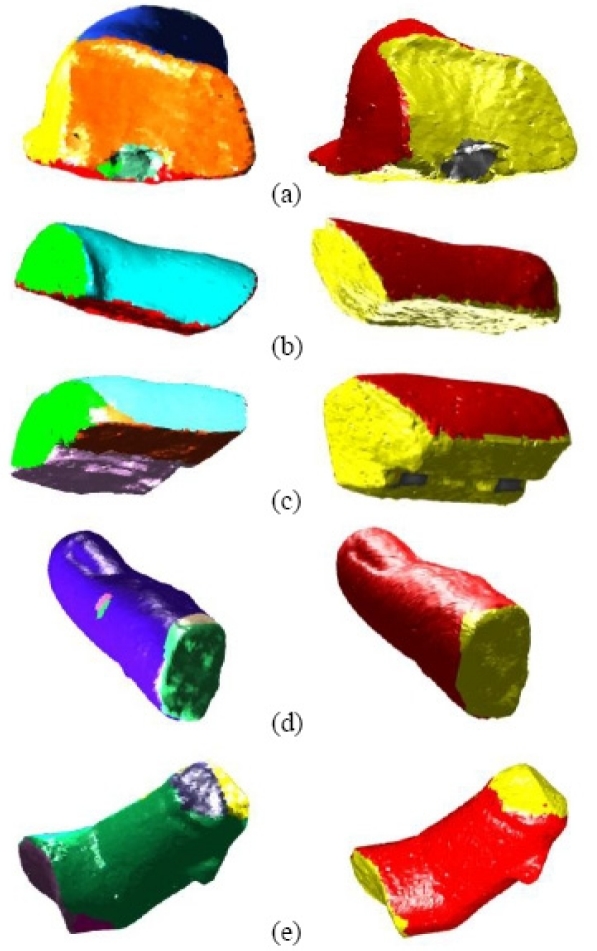
Results of the segmentation and characterization algorithm for five pieces (**a**) to (**e**). **Left:** The faces extracted after the 3D segmentation phase in different colors. **Right:** The original faces of the fragments are shown in red. The yellow zones are fractured parts.

In essence, the segmentation algorithm helps to distinguish between zones of the fragments that belonged to visible parts of the sculpture (the original faces) and those that did not (the fractured faces). Figure 14 shows the results after running the algorithm on some of the generated meshes. This information makes the calculation of the fragments pose onto the virtual model easier.

The following stage in the inference process consists of calculating the positioning of each fragment in the virtual 3D model corresponding to the part of the body assigned by historians. Note that the original labeled parts of a fragment should be completely matched in the partial virtual model. The matching process was carried out following the algorithm published by Zhang in [24]. The overall registration error is then evaluated and a sorted list of poses is obtained. The best geometrical registration is eventually validated by considering the lowest value of the registration error. This process is performed for all fragments. A consistency algorithm globally evaluates the integration of all fragments into the current virtual model and detects incoherent poses. For example, incoherence cases occur when two of more fragments are partially overlapped onto the model. The consistency algorithm then updates the list of the poses until a global consistency is achieved.

After this, the computational results are then evaluated by historians. They can thus make decisions about altering or not the initial hypotheses. For example, they might modify the virtual model or update the set of assignments of the fragments to the set of partial models. This process iterates until the pose of all fragments is satisfactory.

The final stage consists of making a refinement of the model in order to give a more realistic look to the sculptural group. Essentially, this process is carried out by the modeler including, for example, contemporary clothes and minor details.

**Figure 15.**
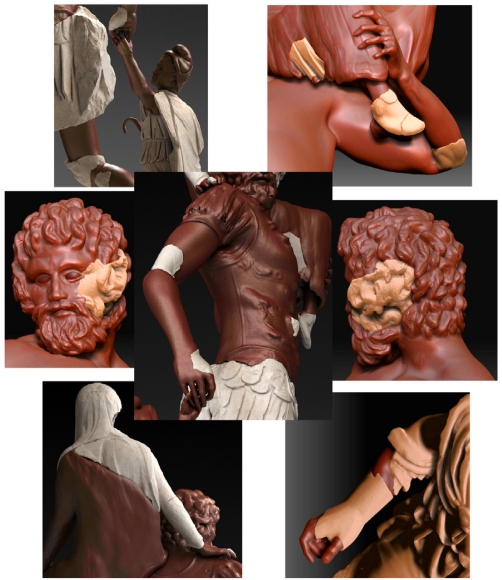
Details of the virtual hypothetical model with recovered fragments coupled into it.

**Figure 16.**
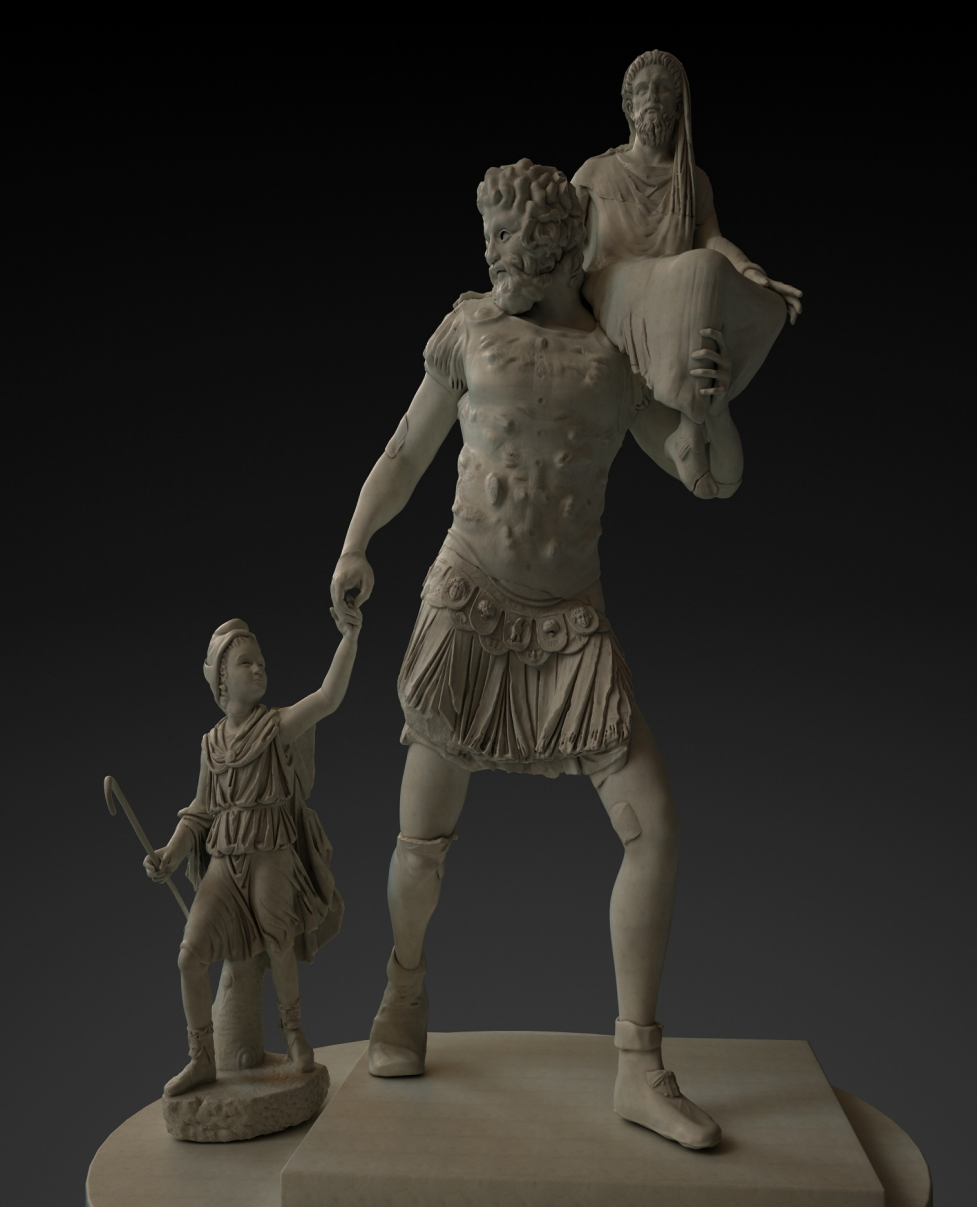
Rendered representation of our proposal for the Roman masterpiece called Aeneas Group.

Figures 1, 15 and 16 show our final proposal for the position of the recovered pieces and their integration in the virtual model generated. Although the reconstruction of the Aeneas Group is now finished, we are continuing to work on our method to give more detail to the surface characterization, with the purpose of being able to solve this type of puzzle with a minimum of user interaction.

The final result of this cross-discipline collaborative research project was presented in a monographic exhibition in the MNAR, in which the full set of objectives attained were shown. One of them, that is particularly worth mentioning, was the development of visualization software as a tool for researchers in history and archaeology. The following subsection will briefly describe this development.

### 4.2. Interactive Viewer

Beyond the computer graphics and computer vision research involved in cultural heritage 3D modelling, the interdisciplinary aspects and social benefits that can come out of this type of collaboration are outstanding. The work that we did during this project led to some applications that our historian and archaeologist colleagues found both useful and with a potential for facilitating the dissemination of knowledge of this period of history. The most noteworthy, of course, was a solution for the positions of fragments on a virtual representation of the Aeneas Group.

We considered that our target audience will probably include users who are unfamiliar with interactive 3D graphics software, and that art historians will not find a large digital model very useful. We therefore developed an easy-to-use visualization computer application bearing in mind the possible problems in the combination of a large data set and a relatively slow computer. This required us to pay special attention to the trade-offs between speed and quality, and between usability and flexibility. To this end, we adopted some appropriate strategies for the movement and visualization of very dense mesh processes, multi-window management, data organization, and handling of the video adapter, which lightened the computational load and improved the performance of the software.

**Figure 17.**
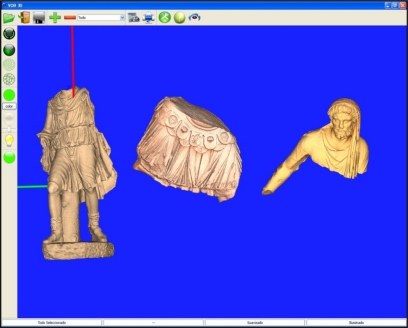
Screen-capture of the interactive viewer.

The interactive viewer can handle *wrl* and *vir* formats. The *vir* format is a proprietary format created to safeguard the copyright of the models, which stores information on faces, vertices, and textures. This software is currently being used by historians and archaeologists in the National Museum of Roman Art. Functions that can change the original 3D meshes have not been implemented, since, as was mentioned above, our users are not accustomed to this type of tool. However, other actions related to display, illumination, and elementary measurements are easy to perform. The software also permits several models to be loaded into the same window with the option of moving them around, and allows multiple windows to be open at the same time. Having a virtual model of the statues lets researchers examine them at their leisure, perhaps even discover details they had not noticed in the time they spent with the physical statue, and check their recollections and theories. The ability to precisely control the lighting lets them see the statue as it might have appeared in environments outside the museum, or highlight small details that are not easily visible. Measuring the statue freely and precisely lets historians factor out subjective perceptions, and better understand the artist's use of perspective and composition. Figure 17 is a screen-grab of the interactive viewer with the final models of the three large pieces loaded.

## 5. Conclusions

In this paper we have presented a method to obtain a 3D geometrical, textural, and physical reconstruction of ancient sculptural groups. In particular, a Roman sculptural group dating from the first century B.C., the Aeneas Group, is restituted The complex shapes in the geometry and the material of the pieces made the digitalization process quite difficult.

One part of the paper is devoted to showing a new strategy with which to generate models of ancient fragments. We briefly show how an accuracy geometry + color model of the existing fragments using a laser scanner is obtained. In this process we include some details of a new filling holes algorithm developed. We also deal with the segmentation of the fragment and the labeling of the segmented regions. The motivation for carrying out new segmentation algorithms lies in the fact that standard local feature based approaches are clearly inefficient in the segmentation of ancient fractured pieces which have suffered centuries of erosion. The pose calculation of the fractured pieces in the virtual model is solved by using an original feed-forward matching algorithm which eventually converges by imposing an overall matching evaluation.

A proposed solution for the restitution of the Aeneas Group was one of the outcomes of the interdisciplinary nature of this research. This is a major achievement in the field of archaeology which could have worldwide repercussions. Also, the set of tools and applications generated during the work included an interactive viewer specifically designed for historical and archaeological research.

Future work is addressed towards developing efficient intelligent algorithms to aid archaeologists reconstruct incomplete 3D puzzles. In this sense, we aim to extend and improve the current semi-automatic solution, and provide an expert system based on fuzzy logic which is able to propose a limited number of solutions which can then be evaluated by expert historians and archaeologists.

## Figures and Tables

**Table 1.** Details of the data acquisition stage.

**Sculpture**	***Lens (focal) (mm)***	***Distance (cm)***	***Area (cm^2^)***

**Aeneas**	Middle (14)	70–100	26 × 16
**Aeneas belt**	Tele (25)	70–90	11 × 8
**Anchises**	Middle (14)	90–120	30 × 20
**Ascanius**	Wide (8)	70–100	45 × 35

**Table 2.** Data for the geometrical models of the three big pieces.

**Sculpture**	***Views***	***Nodes max.***	***Memory Size (MB)***	***Surface (m^2^)***	***Volume (m^3^)***

**Aeneas**	183	600,253	27.6	2.1331	0.168978
**Anchises**	129	1,624,000	55.4	1.0297	0.033755
**Ascanius**	165	742,786	29	3.1822	0.125176

**Table 3.** Data for the geometrical models of the small fragments.

***#***	***Views***	***Faces max.***	***Memory Size (MB)***	***Surface (cm^2^)***	***Volume (cm^3^)***	***Faces/mm^2^***

**1**	10	877,206	71	1,465.51	3,714.57	5.98
**2**	12	422,240	34	502.82	723.71	8.39
**3**	12	242,924	19.3	489.41	626.18	4.96
**4**	8	234,214	18.6	525.73	762.51	4.45
**5**	10	16,102	18.6	34.1	13.7	4.72
**6**	12	37,536	2.09	61.32	31.87	6.12
**7**	12	66,716	5.22	79.25	112. 18	8.41
**8**	12	87,494	3.82	126.26	75.64	6.92
**9**	9	431,162	34.7	604.82	1,026.46	7.12
**10**	15	172,308	13.6	254	231.33	6.78
**11**	12	127,842	10	190.9	192.32	6.69
**12**	11	371,010	29.8	568.73	859.24	6.52
**13**	11	353,144	18.3	517.94	821.4	6.81
**14**	8	166,050	13	273.52	171.21	6.07
**15**	12	88,618	6.96	148.02	88.54	5.983
**16**	12	265,082	21.1	408.11	556.48	6.49
**17**	12	187,728	14.8	283.9	301.1	6.61
**18**	12	310,264	24.8	443	556.27	7.00
**19**	12	308,132	24.6	451.54	573.52	6.8
**20**	12	310,264	6.30	132.28	143.37	23.45
**21**	15	99,562	7.84	167.88	118.58	5.93
**22**	18	689,666	56	1,412.67	2,677.16	4.88
**23**	11	270,430	21.9	535.33	421.05	5.054
**24**	7	384,016	30.9	910.57	971.08	4.21
